# Improving Video Segmentation by Fusing Depth Cues and the Visual Background Extractor (ViBe) Algorithm

**DOI:** 10.3390/s17051177

**Published:** 2017-05-21

**Authors:** Xiaoqin Zhou, Xiaofeng Liu, Aimin Jiang, Bin Yan, Chenguang Yang

**Affiliations:** 1 College of Computer and Information Engineering, Hohai University, Nanjing 211100, China; zhouxq@hhu.edu.cn; 2 Changzhou Key Laboratory of Robotics and Intelligent Technology, Changzhou 213022, China; jiangam@hhuc.edu.cn; 3 Jiangsu Key Laboratory of Special Robots, Hohai University, Changzhou 213022, China; 4 College of IoT Engineering, Hohai University, Changzhou 213022, China; 5 College of Electronics, Communication and Physics, Shandong University of Science and Technology, Qingdao 266590, China; yanbinhit@hotmail.com; 6 Zienkiewicz Centre for Computational Engineering, Swansea University, Swansea SA1 8EN, UK; cyang@theiet.org

**Keywords:** object detection, background subtraction, video surveillance, Kinect sensor fusion

## Abstract

Depth-sensing technology has led to broad applications of inexpensive depth cameras that can capture human motion and scenes in three-dimensional space. Background subtraction algorithms can be improved by fusing color and depth cues, thereby allowing many issues encountered in classical color segmentation to be solved. In this paper, we propose a new fusion method that combines depth and color information for foreground segmentation based on an advanced color-based algorithm. First, a background model and a depth model are developed. Then, based on these models, we propose a new updating strategy that can eliminate ghosting and black shadows almost completely. Extensive experiments have been performed to compare the proposed algorithm with other, conventional RGB-D (Red-Green-Blue and Depth) algorithms. The experimental results suggest that our method extracts foregrounds with higher effectiveness and efficiency.

## 1. Introduction

In recent years, enormous amounts of data on human or animal behavior have been collected using 2D and 3D cameras, and automated methods for detecting and tracking individuals or animals have begun to play an important role in studies of experimental biology, behavioral science, and related disciplines. Tracking target is prone to being lost due to managing appearance changes, fast motion, and other factors. This results in the problem of detecting the tracking target again. Traditional detection methods (such as the detector in TLD (Tracking-Learning-Detection) [1]) treat every frame independently and perform full scanning of an input frame to localize all appearances that have been observed and learned in the past. It is inefficient and time consuming, especially for real-time tracking. However, foreground segmentation requires a detector to scan only the foreground region of an input frame using a scanning window, which greatly reduces the scanning time of the detector and also improves the classification accuracy. At present, a variety of foreground extraction methods have been proposed, such as the frame difference method [2], the background subtraction method [3], the optical flow method [4] and the block matching method [5]. The core of a background subtraction algorithm is the modeling of the background. Zones that show notable differences between the current frame and the background model are deemed to correspond to moving objects. Generally, background subtraction algorithms include the Average Background Model (AVG) algorithm, the Gaussian Mixture Model (GMM) algorithm [6], the Codebook algorithm [7] and the Visual Background Extractor (ViBe) algorithm [8,9,10]. The ViBe algorithm is a fast motion detection algorithm proposed by Olivier Barnich et al. [8]. It is characterized by a high processing efficiency and a good detection effect.

Most of the conventional methods mentioned above were designed for application to color images. However, depth is another interesting cue for segmentation that is less strongly affected by the adverse effects encountered in classical color segmentation, such as shadow and highlight regions. Depth cameras, such as the Microsoft Kinect and the ASUS Xtion Pro (ASUS, Taiwan), are able to record real-time depth video together with color video. Because of their beneficial depth imaging features and moderate price, such depth cameras are broadly applied in intelligent surveillance, medical diagnostics, and human–computer interaction applications [11,12,13]. The Kinect sensor is not sensitive to light conditions; it works well either in a bright room or in a pitch black one. This makes depth images more reliable and easier for a computer program to understand.

Most studies using the Kinect sensor have focused on human body detection and tracking [14,15,16]. The Histogram of Oriented Depths (HOD) detection algorithm, proposed in [14], can be used to match human body contour information in an image. In [15], a model was presented for detecting humans using a 2D head contour model and a 3D head surface model. In these studies, the computational complexity of the feature generation and matching process was relatively high.

**Figure 1:**
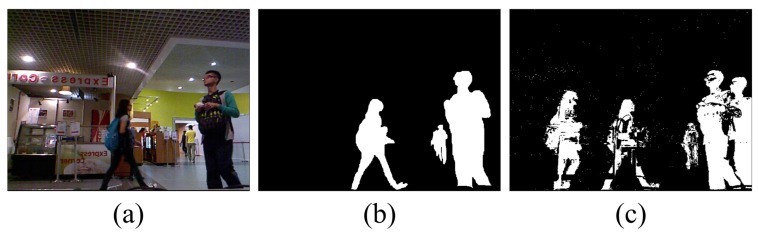
Ghosting in a foreground segmentation map generated by the Visual Background Extractor (ViBe) algorithm [8]: (**a**) color frame; (**b**) ground truth; (**c**) foreground extraction result.

Crabb et al. [17] and Schiller et al. [18] focused on combining the depth and color information obtained by low-resolution ToF (Time of Flight) cameras, but their methods are not well suited for video surveillance. For example, the method of Crabb et al. [17] requires the definition of a distance plane where no foreground object is located behind any part of the background. Fernandez-Sanchez et al. [19] proposed an adaptation of the Codebook background subtraction algorithm that focuses on different sensor channels. In [20], the method presented combines a mixture of Gaussian-based background subtraction algorithms with a new Bayesian network. The Bayesian network exploits the characteristics of the depth data using two dynamic models that estimate the spatial and depth evolution of the foreground/background regions. Camplani et al. [21] proposed a foreground/background segmentation method that combines two statistical classifiers using color and depth features. The combination of depth and color cues makes it possible to solve color segmentation issues such as shadows, reflections and camouflage. However, the computations required are too complicated. The fundamental idea of the combination strategy in [22] is that when depth measurement is reliable, the background subtraction from depth takes top priority. Otherwise, RGB (Red-Green-Blue) is used as an alternative. Noise is removed from the depth data using a noise model. They define the background as the stationary part of a scene. The Gaussian mixture model is observed for each pixel over a sequence of frames. These existing RGB-D (RGB and Depth) segmentation algorithms either suffer from ghosts, such as Depth-Extended Codebook ( DECB) [19] and 4D version of Mixture of Gaussians (MOG4D) [18], or fail to achieve real-time performance [21]. A ghost is a set of interconnected points that is detected as a moving object but does not correspond to any real object (see Figure 1c). Ghosting greatly reduces the effectiveness of motion detection.

In this paper, we propose an adaptive ViBe background subtraction algorithm that fuses the depth and color information obtained by the Kinect sensor to segment foreground regions. First, a background model and a depth model are established. Then, based on these models, we develop a new updating strategy that can efficiently eliminate ghosting and black shadows. The improved algorithm is evaluated using an RGB-D benchmark dataset [23] in addition to our own test RGB-D video and achieves good results that provide a perfect basis for subsequent feature extraction and behavior recognition.

The remainder of the paper is organized as follows. In Section 2, we briefly describe the original ViBe algorithm. Then, the improved algorithm is developed in Section 3. In Section 4, experimental results and discussions are presented. Finally, we conclude the paper in Section 5.

## 2. ViBe Background Subtraction Algorithm

In this section, we first review the basic ViBe algorithm. Then, we identify its disadvantages. This technique involves modeling the background based on a set of samples for each pixel. New frames are compared with the background model, pixel by pixel, to determine whether each pixel belongs to the background or the foreground.

### 2.1. Pixel Model

Background model construction begins from the first frame. Formally, let v(x) denote the value in a given Euclidean color space associated with the pixel located at *x* in the image, and let $v_{i}$ be the background sample value with index *i*. Each background pixel *x* is modeled based on a collection of *N* background sample values $M\left(x\right)\;=\left\{v_{1},v_{2},\cdots ,v_{N}\right\}$.

### 2.2. Classification Process

If the Euclidean distance from a sample $v_{i}$ in M(x) to v(x) is below a threshold *R*, then $v_{i}$ is regarded as a neighbor of v(x). We define the number of neighbors of the pixel located at *x* as $N_{R}\left(x\right)=\left\{∥v\left(x\right)-v_{i}∥<R,\;\forall v_{i}\in M\left(x\right)\right\}$. When $N_{R}\left(x\right)$ is greater than a threshold λ, *x* is a background pixel. Otherwise, it is a foreground pixel.

### 2.3. Updating the Background Model over Time

It is necessary to continuously update the background model with each new frame. This is a crucial step for achieving accurate results over time. When a pixel *x* is classified as background, the background model updating strategy is triggered. A sample is chosen randomly. Mathematically, the probability that a sample present in the model at time *t* will be preserved is given by (N−1)/N. Under the assumption of time continuity, for any later time t+dt, this probability is equal to:

(1) $$P\left(t,t+dt\right)={\left(\frac{N-1}{N}\right)}^{(t+dt)-t}$$

which can be rewritten as:

(2) $$P\left(t,t+dt\right)=e^{-ln{\left(\frac{N}{N-1}\right)}^{dt}}$$

This expression shows that the expected remaining lifespan of any sample value in the model decays exponentially according to a random subsampling strategy. As in [8], we adopt a time subsampling factor of ϕ, meaning that a background pixel value has one chance in ϕ of being selected to update its pixel model.

Reduced pseudo-code for the ViBe construction phase is given in Algorithm 1.

The classical ViBe algorithm has the advantages of simple processing and outstanding performance. Its main drawback is the occurrence of ghosting. A moving object in the first frame often causes ghosting. To resolve this problem, we can take advantage of depth information. The Kinect sensor records the distance to any object that is placed in front of it. This feature can be utilized to determine whether a foreground pixel is a ghost.

**Algorithm 1** Algorithm for ViBe construction
  1:**procedure** ViBe(image, *N*, *R*, λ, ϕ)  2:      **for** each pixel **do**  3:            **while** matches<λ and index<N **do**  4:                 Calculate Euclidean distance between $v_{x}$ and $v_{i}$  5:                 **if** dist<R **then**  6:                     matches←matches+1  7:                 **end if**  8:                 index←index+1  9:            **end while**10:            **if** matches⩾λ **then**11:                 Store that pixel ∈ background12:                 Update current pixel background model with probability 1/ϕ13:                 Update neighboring pixel background model with probability 1/ϕ14:            **else**15:                 Store that pixel ∈ foreground16:            **end if**17:      **end for**18:**end procedure**


## 3. Fusion: Depth-Extended ViBe (DEVB)

### 3.1. Depth Model

In general, the first frame in a video serves as the background. When the target appears in the first frame, most of detection methods get difficult to dissociate the foreground from the background quickly only by means of RGB images. However, it would become easy when exploiting depth cues. For a depth image, a pixel is brighter and its distance to camera is closer. Accordingly, the pixel values of a foreground are large, while those of a background are small. When a foreground shifts, the pixels corresponding to its initial position naturally get small. If those pixels are still recognized as the foreground, they would be a false foreground (a ghost).

To eliminate ghosting, the ViBe algorithm is improved by enhancing the matching conditions when a pixel is classified as foreground. A depth model $M_{D}\left(x\right)$ is added. Initially, the pixel values of the first depth frame are saved to $M_{D}\left(x\right)$. This depth model also has an updating strategy similar to that for the background model. When the updating strategy is triggered, the depth value $M_{D}\left(x\right)$ is replaced with that corresponding to the current pixel. If the following condition is satisfied, this pixel will be considered a ghost pixel:

(3) $$v\left(x,t_{0}\right)-v\left(x,t\right)>\tau$$

where $v(x,t_{0})$ is the value of the pixel located at *x* in the depth model $M_{D}\left(x\right)$ at time $t_{0}$, v(x,t) is the value of the pixel located at *x* in the current depth image at time *t*. The threshold τ is an empirical value, which can be tuned to obtain the best result. The value of τ is recommended to lie within the range of $\left[1,3\right]$.

### 3.2. Fusion Algorithm for Color and Depth Images

A color image conforms to an individual’s visual habits and provides detailed information such as color and texture. The most intuitive fusion strategy is to add the depth information as a fourth channel to the ViBe algorithm for color images. The channel combination $f=(f_{r},f_{g},f_{b},d)$ is formed from the three color channels in RGB space and the depth value *d*. In this way, we intend to utilize depth information as a measure of reliability during segmentation. A depth image quantifies the distance from an object to the camera. The higher the value of a pixel is, the more reliable its measurement is in the depth image. Thus, we use the inverse of the depth image to allow it to be used in the same way as a variance image.

The inverted depth values are normalized between zero and one, and the normalized uncertainty is denoted by σ(x). In a region where the depth uncertainty is high, the depth measurement is considered unreliable. For example, where there are holes in the depth image, the fusion result will depend on the color image. We weight the normalized depth $\hat{d(x)}$ with the uncertainty σ(x), resulting in $w_{d}$ values that range between zero and one depending on σ(x). Meanwhile, the color value $\hat{c(x)}$ is multiplied by σ(x) and added to itself to obtain the weighted color value, defined as $w_{c}$. The combined normalized image is denoted by $\hat{I(x)}$, as shown in Equation (4).

(4) $$\left\{\begin{array}{cc} w_{d} & =\left(1-\sigma \left(x\right)\right)\hat{d(x)}\\ w_{c} & =\left(1+\sigma \left(x\right)\right)\hat{c(x)}\\ \hat{I(x)} & =\frac{1}{2}\left(w_{d}+w_{c}\right) \end{array}\right.$$

The original algorithm can eliminate ghosts and black shadows in subsequent frames, but the process is relatively slow. We propose a fusion strategy that incorporates an additional depth model to effectively remove ghosting and black shadows. Reduced pseudo-code for the DEVB construction phase is given in Algorithm 2.

**Algorithm 2** Algorithm for DEVB construction
  1:**procedure** DEVB($image_{fusion}$, $image_{depth}$, *N*, *R*, λ, ϕ)  2:      **for** each $image_{fusion}$ pixel **do**3:            **while** matches<λ and index<N **do**  4:               Calculate Euclidean distance between $v_{x}$ and $v_{i}$  5:               **if** dist<R **then**  6:                     matches←matches+1  7:               **end if**  8:               index←index+1  9:            **end while**10:            **if** matches⩾λ **then**11:                 Store current pixel ∈ background12:                 Update current pixel background model in $image_{fusion}$ with probability 1/ϕ13:                 Update neighboring pixel background model in $image_{fusion}$ with probability 1/ϕ14:                 Update current pixel $model_{depth}$ using $image_{depth}$15:            **else**16:                 Store current pixel ∈ foreground17:                 Find ghosts, calculate Euclidean distances between pixels at the same position in $image_{depth}$ and $model_{depth}$18:                 **if** dist>τ **then**19:                     Store that pixel ∈ background20:                     Update current pixel background model in $image_{fusion}$ with probability 1/ϕ21:                     Update neighboring pixel background model in $image_{fusion}$ with probability 1/ϕ22:                 **end if**23:            **end if**24:      **end for**25:**end procedure**


Section 4 presents experiments performed without post-processing and the results obtained using three RGB-D algorithms (MOG4D [18], DECB [19] and DEVB) as well as the color-based ViBe algorithm.

## 4. Experiments and Results

The program development environment consisted of VC++2010, OpenCV SDK2.4.3 and OpenNI1.5.2.7. The PC was equipped with a Core Duo 2 CPU E7500 with 2.00 GB of RAM. The video frame rate was 30 fps, and the size of both the color and depth images was 640 × 480. We compared the results of our method with those obtained using two state-of-the-art RGB-D fusion-based background subtraction algorithms, namely, MOG4D [18] and DECB [19], as well as the ViBe algorithm on the color images [8] (ViBe) and the ViBe based only on depth (ViBe1D). To evaluate these algorithms objectively through a quantitative analysis, we required a benchmark that would provide information on both color and depth images. The chosen benchmark sequences are publicly available at [23]. However, the depth image sequences provided by this source could not be utilized directly. The depth images are in the 16-bit png format, with the first three bits swapped with the last. We needed to swap them back after reading each image to obtain values for each pixel corresponding to the distance from the Kinect sensor to the object in mm. The sequences child_no1, new_ex_occ4, walking_occ1 and new_ex_no_occ from [23] and our own pigeon were chosen for testing.

Many metrics can be used to assess the output of a background subtraction algorithm given a series of ground truths for several frames in each sequence. Various relative metrics can be calculated based on the numbers of true and false positives and negatives (TP, FP, TN, and FN). These metrics are most widely used in computer vision to assess the performance of a binary classifier, as in [24]. PWC is the percentage of wrong classifications in the entire image. This measure represents a trade-off between the abilities of an algorithm to detect foreground and background pixels. In general, a lower value of this estimator indicates better performance.

(5) $$PWC=\frac{FN+FP}{TP+TN+FP+FN}\times \;100$$

**Figure 2:**
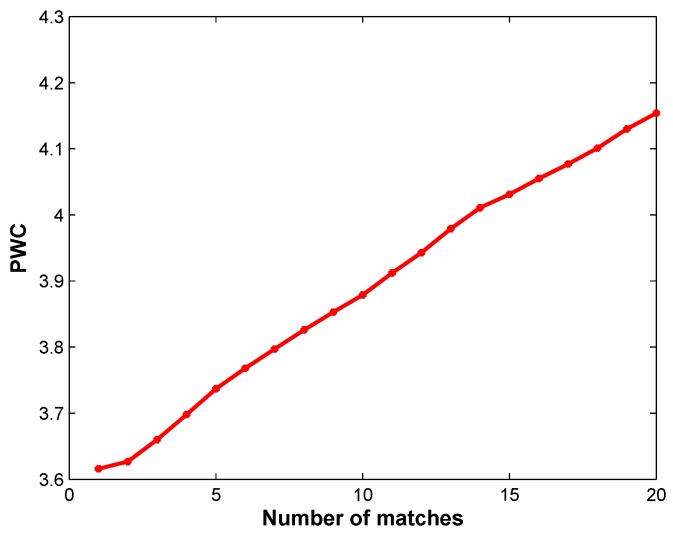
Percentage of wrong classifications (PWCs) for λ ranging from 1 to 20. The other parameters of depth-extended ViBe (DEVB) were set to N=20,R=20, and ϕ=16.

**Figure 3:**
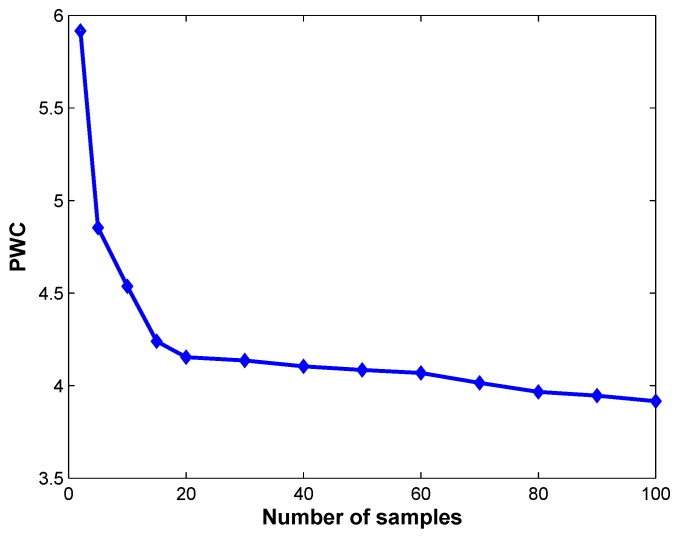
PWCs given the number of samples collected in a background model.

The proposed approach relies on several parameters originating from the ViBe algorithm: *N*, *R*, λ, and ϕ in [8]. Considering our aim of evaluating the overall performance of the algorithms, we chose a unique set of parameters that yielded sufficiently good results on the complete dataset. According to [8], and in our experience, the use of a radius R=20 and a time subsampling factor ϕ=16 leads to excellent results in every situation. To determine optimal values for λ, we computed the evolution of the PWC of DEVB on the new_ex_no_occ sequence for λ ranging from 1 to 20. The other parameters were fixed to N=20,R=20, and ϕ=16. Based on joint consideration of the value of λ in [8] and in Figure 2, we set the optimal value of λ to λ=2. As λ rises, the computational cost increases. Once the value of 2 has been selected for λ, we study the influence of the parameter *N* on the performance of DEVB. Figure 3 shows the percentages obtained using the new_ex_no_occ sequence for *N* ranging from 2 to 50. We observe that higher values of *N* provide better performance. However, they tend to saturate for values higher than 20. We select N at the beginning of the basin, that is, N=20. To ensure a fair comparison of the performances of the various algorithms, all algorithms were applied without morphological filtering.

**Figure 4:**
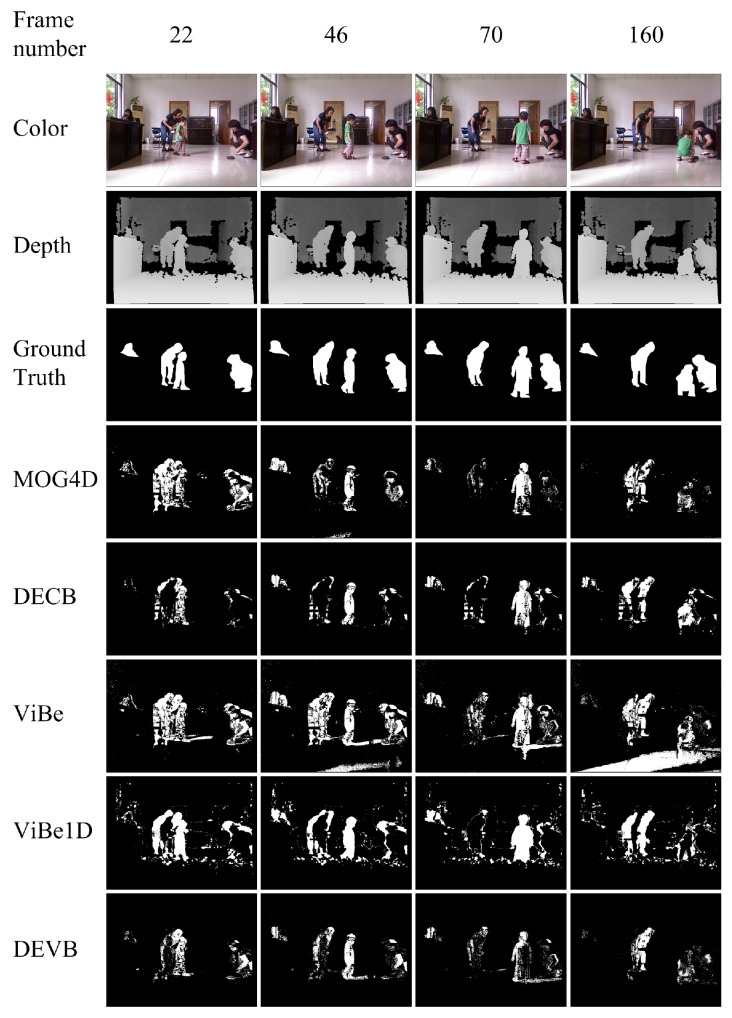
Comparison of background/foreground segmentation images generated by various background subtraction techniques for four frames taken from the child_no1 sequence without morphological filtering. The segmented images produced by our method are the closest to the ground-truth references. MOG4D: 4D version of Mixture of Gaussians. DECB: Depth-Extended Codebook. ViBe: visual background extractor. ViBe1D: ViBe based only on depth. DEVB: Depth-Extended ViBe.

The first sequence, child_no1, shows a child and two adults playing in a living room. The main difficulties in this sequence are light reflections and subjects that sometimes remain still or move only slowly. Figure 4 shows the segmentations produced by the four methods as well as the original color and depth frames and the hand-generated segmentations (ground truths). Ghosts appear in some frames for MOG4D, DECB, ViBe and ViBe1D, greatly reducing the effectiveness of foreground detection. The ViBe algorithm yields worse results than the other algorithms in frame 160 because of the reflection in the color image. In general, the DEVB algorithm achieves improvement over ViBe by virtue of the additional depth model, which allows the ghosts and black shadows to be effectively removed.

Table 1 shows the quantitative PWC results obtained by the four approaches on the evaluation frames from the child_no1 sequence. All RGB-D approaches achieve improvements with respect to ViBe, obtaining lower PWC values. A lower PWC value indicates better performance. The proposed DEVB algorithm achieves the lowest average error rate of 7.023% in Table 1, which indicates that our method performs better than the other algorithms.

**Figure 5:**
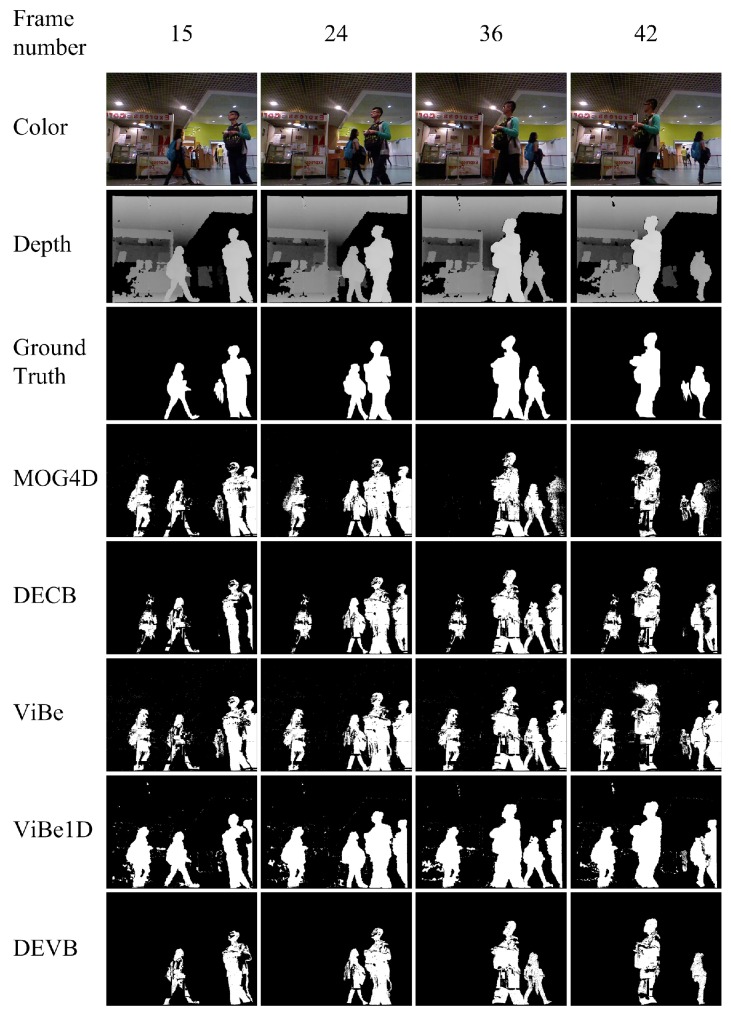
Results obtained in the test sequence new_ex_occ4.

The second sequence, new_ex_occ4, shows two individuals walking in front of a coffee shop. The main difficulties presented by this sequence are flickering lights and areas where depth information cannot be obtained by the active infrared sensor. Figure 5 shows the segmentations produced by the four approaches. Our DEVB algorithm achieves good results, whereas ghosting greatly reduces the effectiveness of the other algorithms in foreground extraction.

Table 2 shows the quantitative PWC results obtained by the four approaches on the evaluation frames from the new_ex_occ4 sequence. The proposed DEVB algorithm achieves the lowest PWC of 3.968, which indicates that our method performs better than the other algorithms. ViBe yields the worst result of PWC=12.872 on this sequence.

**Figure 6:**
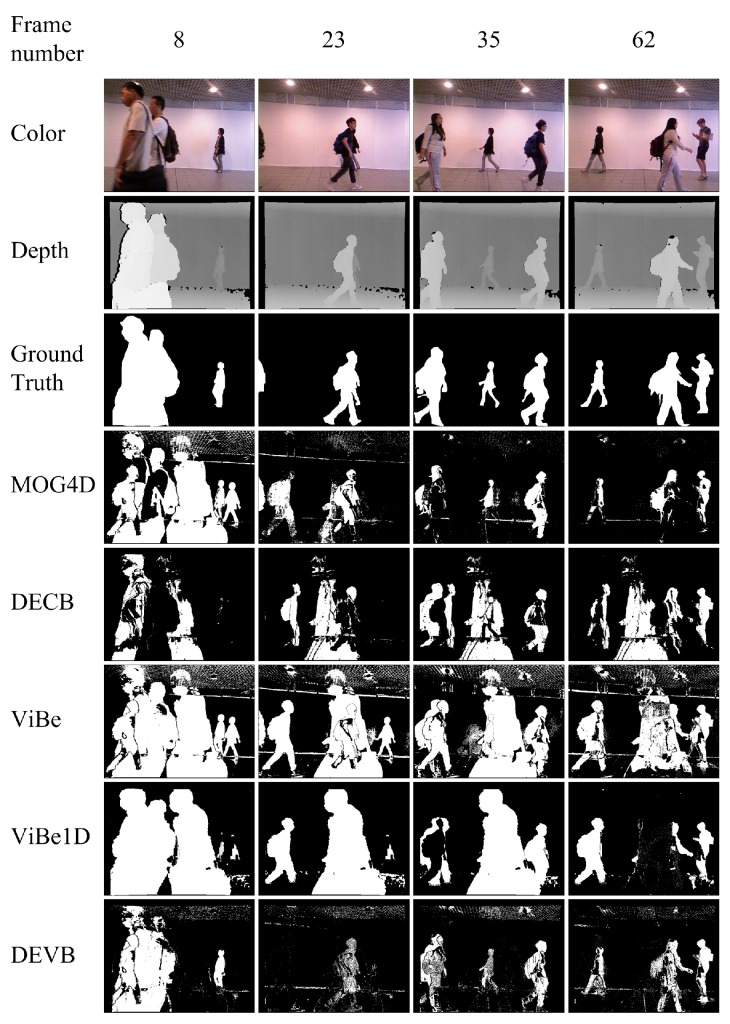
Results obtained in the test sequence walking_occ1.

The third sequence, walking_occ1, shows a few people walking in and out of the camera field. In addition, there are flickering lights on the ceiling and sudden illumination changes. Figure 6 shows the segmentations produced by the four approaches. DECB and ViBe1D are less affected by the sudden illumination changes. In addition to ghosting, ViBe1D results in black shadows in frames 35 and 62. The reason for the generation of black shadows is that a new moving object reaches the previous position of an old target. Because the pixel values are similar, the foreground is misclassified as background.

Table 3 shows the quantitative PWC results obtained by the four approaches on the evaluation frames from the walking_occ1 sequence. The DEVB algorithm achieves PWC=8.847 in frame 8, whereas the PWC values obtained by the other algorithms are higher by more than a factor of three. Moreover, despite being affected by illumination changes in the RGB space, DEVB achieves an average PWC of 8.721 (Table 3), indicating that our method is fairly robust to difficult situations.

**Figure 7:**
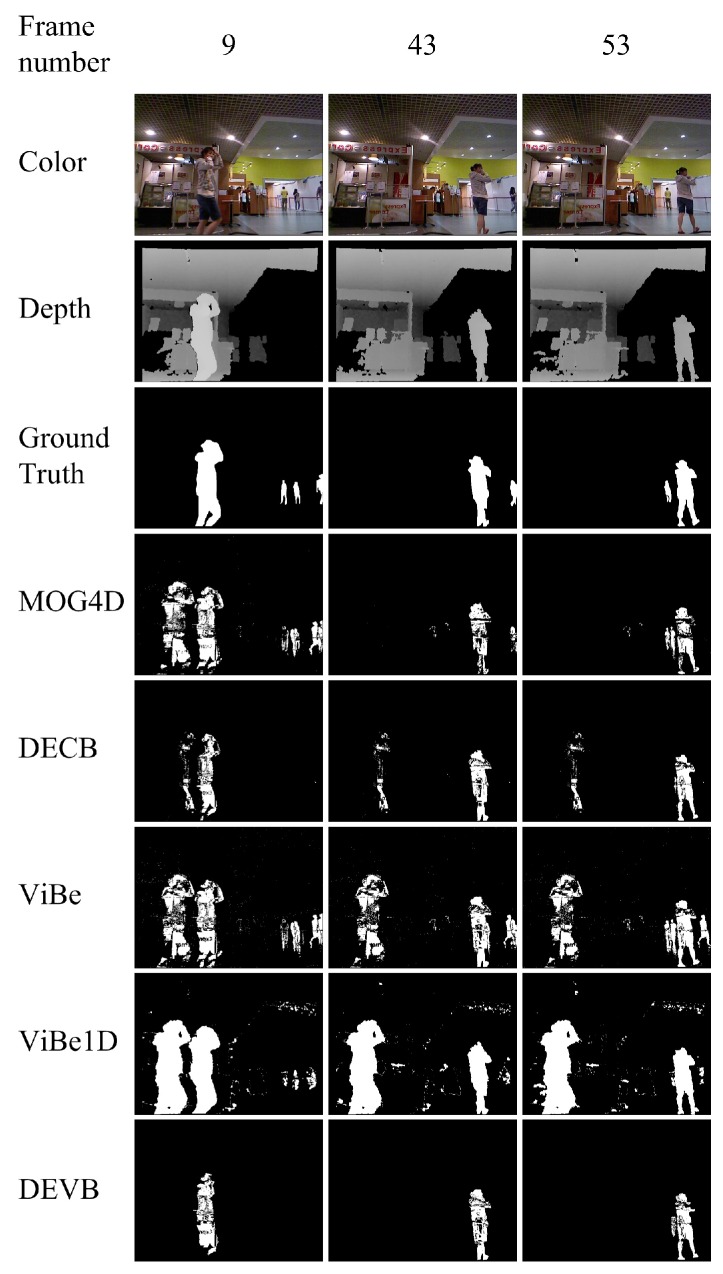
Results obtained in the test sequence new_ex_no_occ.

The fourth sequence, new_ex_no_occ, shows a lady walking in front of a coffee shop. The scenario is similar to the new_ex_occ4 sequence discussed above. Figure 7 shows the segmentations produced by the four approaches. A large amount of noise is generated by the ViBe1D algorithm because of the holes in the original depth image.

Table 4 shows the quantitative PWC results obtained by the four approaches on the evaluation frames from the new_ex_no_occ sequence. The proposed DEVB algorithm achieves PWC=2.437. This value is the lowest in Table 4, which indicates that our method performs better than the other algorithms. ViBe1D obtains the worst result in this sequence, with PWC=12.389.

**Figure 8:**
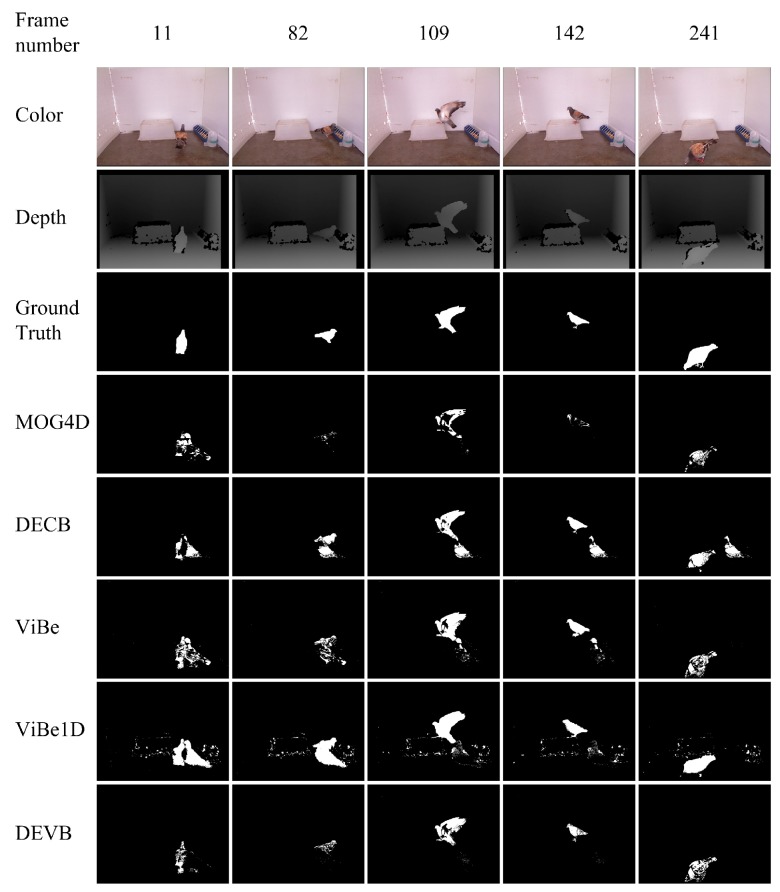
Results obtained in the test sequence pigeon.

The fifth sequence, pigeon, is from our own test RGB-D video. The pigeon has a wide range of activities in the scene as shown in Figure 8. It can jump onto a high platform and walk beside a water bottle and a feeder, which serve as the background. Because of the fast and abrupt pose changes of the pigeon, most of the tracking algorithms fail to track in most of the frames. We focus on detection technology and on utilizing the corresponding depth images, which extracts foregrounds more effectively and more efficiently. In addition to ghosting, ViBe1D results in much noise because of the holes in the original depth image. DECB suffers from ghosting in all test frames.

Table 5 shows the quantitative PWC results obtained by the four approaches on the evaluation frames from the pigeon sequence. The proposed DEVB algorithm achieves PWC=0.778. This value is the smallest in Table 5, which indicates that our method performs better than the other algorithms. ViBe1D obtains the worst result on this sequence, with PWC=2.245.

**Figure 9:**
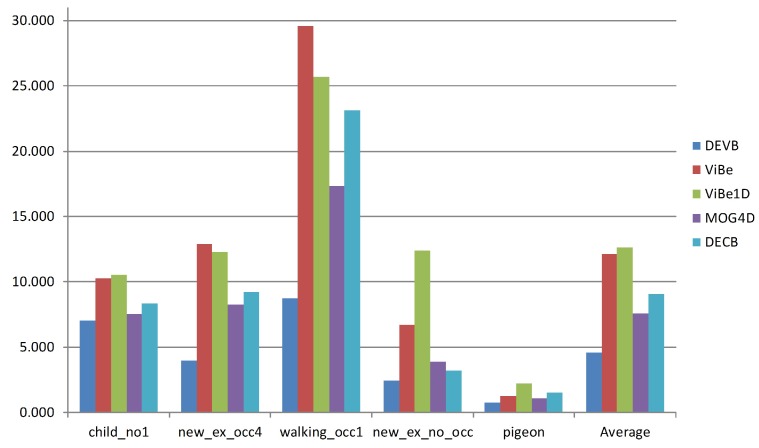
Average PWC values for each of the four sequences and for the entire dataset.

Finally, Figure 9 shows the average PWC value obtained by each approach on each sequence. According to this figure, DEVB yields the best results on every sequence. The walking_occ1 sequence is particularly complicated because of the high pedestrian flow; consequently, for each algorithm, the error rate is considerably increased.

## 5. Conclusions

In this paper, we present an efficient moving object detection algorithm that fuses depth and color information. To incorporate the features of depth images, a depth model is designed in addition to a background model. The inspection mechanism for the classification of foreground pixels is further considered. Finally, we propose a new updating strategy based on the developed background and depth models, which can eliminate ghosting and black shadows almost completely. Experimental results indicate that our method is able to extract foregrounds efficiently, providing an excellent basis for the subsequent motion analysis of a scene. Our proposed method could serve as a convenient research tool for the detection of moving objects captured by the Kinect sensor.

## Figures and Tables

**Table 1:** Segmentation evaluation for the child_no1 sequence. The table shows the PWC results for the various approaches on four different evaluation frames and the mean values for this sequence.

Approach	Frame 22	Frame 46	Frame 70	Frame 160	Mean
DEVB	**6.114**	**6.035**	8.826	**7.118**	**7.023**
ViBe	7.808	8.932	**7.879**	16.455	10.269
ViBe1D	11.183	10.686	9.458	10.848	10.544
MOG4D	7.519	6.559	8.003	8.13	7.553
DECB	8.271	8.072	8.672	8.334	8.337

**Table 2:** Segmentation evaluation for the new_ex_occ4 sequence. The table shows the PWC results for the various approaches on four different evaluation frames as well as the mean values for this sequence.

Approach	Frame 15	Frame 24	Frame 36	Frame 42	Mean
DEVB	**5.129**	**2.997**	**3.261**	**4.485**	**3.968**
ViBe	12.294	12.394	12.684	14.116	12.872
ViBe1D	12.096	12.554	12.461	11.999	12.278
MOG4D	11.685	11.157	4.845	5.189	8.219
DECB	9.753	8.522	8.706	9.909	9.223

**Table 3:** Segmentation evaluation for the walking_occ1 sequence. The table shows the PWC results for the various approaches on four different evaluation frames and the mean values for this sequence.

Approach	Frame 8	Frame 23	Frame 35	Frame 62	Mean
DEVB	**8.847**	**7.728**	**10.17**	**8.137**	**8.721**
ViBe	33.287	30.628	30.221	24.078	29.554
ViBe1D	29.944	26.806	31.726	14.332	25.702
MOG4D	38.186	11.195	10.798	9.185	17.341
DECB	30.262	18.616	22.371	21.162	23.103

**Table 4:** Segmentation evaluation for the new_ex_no_occ sequence. The table shows the PWC results for the various approaches on three different evaluation frames as well as the mean values for this sequence.

Approach	Frame 9	Frame 43	Frame 53	Mean
DEVB	**4.102**	**1.549**	**1.66**	**2.437**
ViBe	8.188	5.832	6.097	6.706
ViBe1D	14.009	11.249	11.91	12.389
MOG4D	7.977	1.719	1.842	3.846
DECB	5.264	2.222	2.225	3.237

**Table 5:** Segmentation evaluation for the pigeon sequence. The table shows the PWC results for the various approaches on five different evaluation frames as well as the mean values for this sequence.

Approach	Frame 11	Frame 82	Frame 109	Frame 142	Frame 241	Mean
DEVB	**0.972**	**0.626**	**0.897**	**0.204**	**1.191**	**0.778**
ViBe	1.837	1.126	1.415	0.603	1.268	1.250
ViBe1D	3.559	3.025	2.156	1.224	1.261	2.245
MOG4D	1.360	0.794	1.222	0.630	1.444	1.090
DECB	1.742	1.157	1.563	1.218	2.029	1.542
